# (1′*S*,6′*S*,8′*S*,9′*R*)-9′-Bromo-12′-oxa­spiro­[1,3-dioxolane-2,4′-tricyclo­[6.3.1.0^1,6^]dodeca­ne]

**DOI:** 10.1107/S1600536812029777

**Published:** 2012-07-10

**Authors:** Goverdhan Mehta, Tabrez Babu Khan

**Affiliations:** a Department of Organic Chemistry, Indian Institute of Science, Bangalore 560 012, Karnataka, India; b School of Chemistry, University of Hyderabad, Hyderabad 500 046, A.P. India

## Abstract

In an endeavor directed towards the construction of the oxabicyclic[3.2.1]octane segment present in the bioactive natural products of cortistatins and icetexanes genre, the title compound, C_13_H_19_BrO_3_, was synthesized from (4a*R*,9a*S*)-1,3,4,4a,5,6,9,9a-octa­hydro­spiro­[benzo[7]annulene-2,2′-[1,3]dioxolane]-4a-ol *via* a transannular bromo-etherification protocol. The six-membered ring adopts a twist-boat conformation, while the fused cycloheptane ring adopts a chair conformation. The crystal packing is effected through two distinct inter­molecular C—H⋯O hydrogen-bond patterns and mol­ecules are arranged to define an inter­esting motif along the *b* axis.

## Related literature

For the isolation and biological activity of cortistatins, see: Aoki *et al.* (2006 ▶, 2007 ▶); Watanabe *et al.* (2007 ▶); Zhao (2010 ▶) and for icetexanes, see: Esquivel *et al.* (1995 ▶); Uchiyama *et al.* (2005 ▶). For synthetic approaches towards the construction of the oxabicyclic core of cortistatins, see: Zhao (2010 ▶); Hardin Narayan *et al.* (2010 ▶) and references cited therein. For their use in the treatment of blindness, see: Czako *et al.* (2009 ▶). For the construction of relevant 6/7 fused-ring systems involving ring-closing metathesis, see: Mehta & Likhite (2008 ▶, 2009 ▶). For an example of the exploitation of transannular bromo­etherification towards natural products synthesis, see: Mehta & Sen (2010 ▶); Mehta & Yaragorla (2011 ▶).
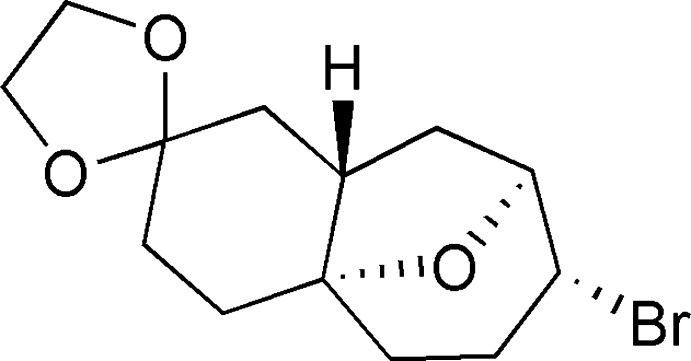


## Experimental

### 

#### Crystal data

C_13_H_19_BrO_3_
*M* *_r_* = 303.19
Monoclinic, 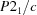

*a* = 11.0159 (3) Å
*b* = 12.6619 (3) Å
*c* = 10.2763 (2) Å
β = 117.044 (1)°
*V* = 1276.63 (5) Å^3^
*Z* = 4
Mo *K*α radiation
μ = 3.21 mm^−1^
*T* = 296 K
0.30 × 0.20 × 0.15 mm

#### Data collection

Bruker APEXII CCD diffractometer
Absorption correction: multi-scan (*SADABS*; Bruker, 2008 ▶) *T*_min_ = 0.446, *T*_max_ = 0.644
11338 measured reflections
2368 independent reflections
1859 reflections with *I* > 2σ(*I*)
*R*_int_ = 0.027

#### Refinement

*R*[*F* ^2^ > 2σ(*F* ^2^)] = 0.030
*wR*(*F* ^2^) = 0.076
*S* = 1.02
2368 reflections
154 parameters
?
Δρ_max_ = 0.25 e Å^−3^
Δρ_min_ = −0.32 e Å^−3^

### 

Data collection: *APEX2* (Bruker, 2008 ▶); cell refinement: *SAINT* (Bruker, 2008 ▶); data reduction: *SAINT*; program(s) used to solve structure: *SHELXS97* (Sheldrick, 2008 ▶); program(s) used to refine structure: *SHELXL97* (Sheldrick, 2008 ▶); molecular graphics: *SHELXTL* (Sheldrick, 2008 ▶); software used to prepare material for publication: *SHELXTL*.

## Supplementary Material

Crystal structure: contains datablock(s) I, global. DOI: 10.1107/S1600536812029777/ds2204sup1.cif

Structure factors: contains datablock(s) I. DOI: 10.1107/S1600536812029777/ds2204Isup2.hkl

Supplementary material file. DOI: 10.1107/S1600536812029777/ds2204Isup3.cml

Additional supplementary materials:  crystallographic information; 3D view; checkCIF report

## Figures and Tables

**Table 1:** Hydrogen-bond geometry (Å, °)

*D*—H⋯*A*	*D*—H	H⋯*A*	*D*⋯*A*	*D*—H⋯*A*
C11—H11⋯O2^i^	0.98	2.53	3.445 (3)	156
C1—H1⋯O1^ii^	0.98	2.57	3.471 (3)	153

Symmetry codes: (i) 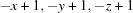; (ii) 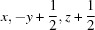.

**Fig. 1.**
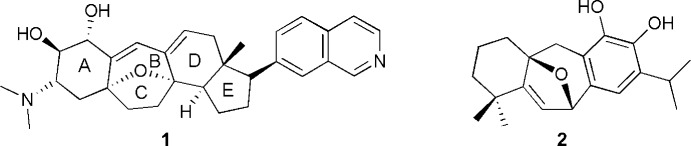
Representative example of cortistatin family (cortistatin A 1) & icetexane family (salviasperanol 2).

**Fig. 2.**
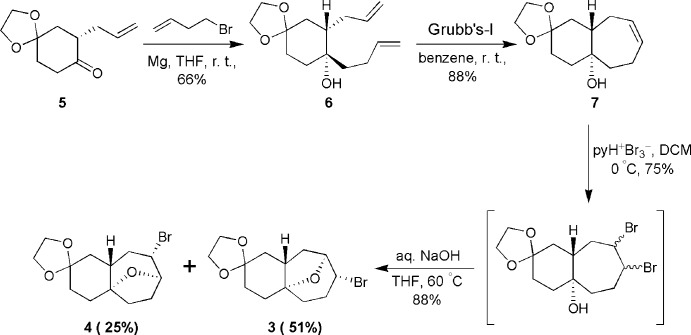
The synthesis of the title compound.

**Fig. 3.**
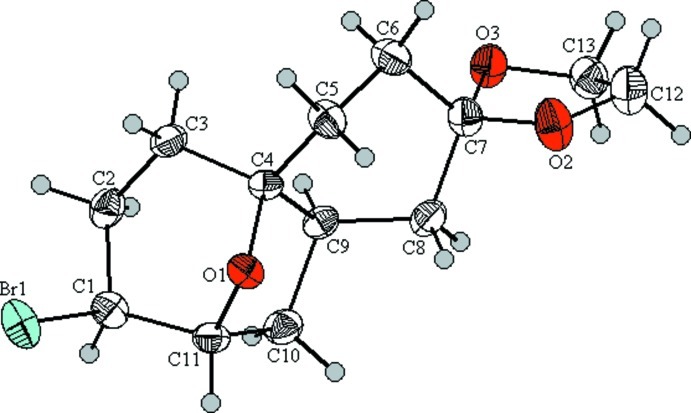
The molecular structure of the title compound 3, with the atom numbering scheme. Dispalcement ellipsoids for non-H atoms are drawn at 30% probability.

**Fig. 4.**
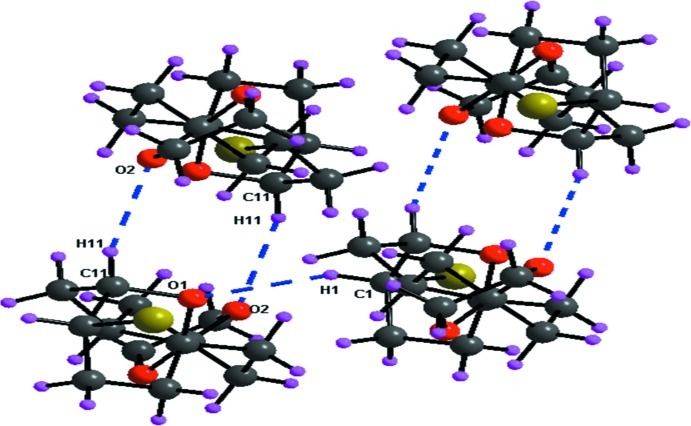
A packing diagram of the title compound 3, viewed along the b axis. Dotted lines indicate the C—H···O hydrogen bonds.

## supplementary crystallographic information

### Comment

The oxabicyclic[3,2,1]octane scaffold manifest itself in a variety of terpenoids class of natural products *viz.* icetexanes (Fig. 1; Esquivel *et al.*, 1995 & Uchiyama *et al.*, 2005) and cortistatin family (Fig. 1; Aoki *et al.*, 2006, 2007 & Watanabe *et al.*, 2007). In particular, cortistatin A and its structural siblings isolated in trace amounts by Kobayashi & coworkers from an Indonesian marine sponge *corticium simplex* were shown to possess novel architecture and exhibited potent and promising anti-angiogenic activity (Zhao, 2010) and were effective in treating blindness (Czako *et al.*, 2009), thereby triggering interest to devise tactics for their total synthesis and diversity creation. These attributes of cortistatins encouraged us to devise a strategy to gain rapid access to the oxatricyclic ABC core present in cortistatins.

Several synthetic approaches to cortistatins have been reported utilizing ring-expansion approach, oxidative dearomatization, electrocyclization, 1,3-dipolar cycloaddition/electrocyclization cascade, transannular [4 + 3] cycloaddition, classical Michael/aldol condensation cascade cyclization as the key strategic steps to access the oxatricyclic segment (Hardin Narayan *et al.*, 2010). However, the present strategy employs a stepwise transannular bromoetherification sequence (Mehta & Sen, 2010; Mehta & Yaragorla, 2011) on a readily accessible bicyclic compound obtained *via* RCM (Fig. 2; Mehta & Likhite, 2008, 2009).

A two step transannular bromoetherification protocol on 7 furnished the title compound 3 (Fig. 2) as the major product corresponding to the oxatricyclic core present in icetexanes along with a minor regioisomeric compound 4 representing the oxatricyclic segment present in cortistatins.

The title compound 3 was crystallized from ethylacetate-hexane(1:1) and the structure was solved and refined in monoclinic *P*2_1_/*c* space group with one molecule of 3 in the asymmetric unit. An *ORTEP* diagram of 3 drawn at 30% ellipsoidal probability is depicted in Fig 3. From the packing diagram it can be seen that the centrosymmetric molecules are connected by weak C11–H11···O2 (2.53 Å, 156°) hydrogen bonds forming a dimeric motif and these dimeric units are further connected by C1–H1···O1 (2.57 Å, 153°)) hydrogen bonds, three dimensionally (Fig. 4).These two hydrogen bond patterns link the molecules to define an interesting motif along the *b* axis.

### Experimental

The synthesis of the title compound 3 as depicted in Fig. 2 emanates from the known 7-(prop-2-en-1-yl)-1,4-dioxaspiro[4.5]decan-8-one 5 through addition of butenylmagnesium bromide (1.5 equiv.) in THF at r.t. to furnish the desired RCM precursor 6 in decent yield. Exposure of 6 to Grubbs-1s t generation catalyst (10 mol%) in benzene at r.t. gave the bicyclic compound 7 in good yield. Finally, the stepwise transannular bromotherification on 7 was executed *via* bromination with pyH^+^Br_3_^-^ (1.2 equiv.) in DCM at 0 °C followed by etherification in the presence of 10 *M* aq. NaOH in THF at 60 °C for 4 h to deliver 3, mp. 78–80 °C, as a colorless crystalline compound in 51% yield.

### Refinement

All the non-hydrogen atoms were refined anisotropically. Hydrogen atoms on the C atoms were introduced on calculated positions and were included in the refinement riding on their respective parent atoms

### Crystal data

**Table:**

C_13_H_19_BrO_3_	*F*(000) = 624
*M**_r_* = 303.19	*D*_x_ = 1.577 Mg m^−^^3^
Monoclinic, *P*2_1_/*c*	Mo *K*α radiation, λ = 0.71073 Å
Hall symbol: -P 2ybc	Cell parameters from 4277 reflections
*a* = 11.0159 (3) Å	θ = 2.6–24.2°
*b* = 12.6619 (3) Å	µ = 3.21 mm^−^^1^
*c* = 10.2763 (2) Å	*T* = 296 K
β = 117.044 (1)°	Block, colorless
*V* = 1276.63 (5) Å^3^	0.30 × 0.20 × 0.15 mm
*Z* = 4	

### Data collection

**Table:**

Bruker APEXII CCD diffractometer	2368 independent reflections
Radiation source: fine-focus sealed tube	1859 reflections with *I* > 2σ(*I*)
Graphite monochromator	*R*_int_ = 0.027
φ and ω scans	θ_max_ = 25.4°, θ_min_ = 2.1°
Absorption correction: multi-scan (*SADABS*; Bruker, 2008)	*h* = −13→12
*T*_min_ = 0.446, *T*_max_ = 0.644	*k* = −15→14
11338 measured reflections	*l* = −9→12

### Refinement

**Table:**

Refinement on *F*^2^	Primary atom site location: structure-invariant direct methods
Least-squares matrix: full	Secondary atom site location: difference Fourier map
*R*[*F*^2^ > 2σ(*F*^2^)] = 0.030	Hydrogen site location: inferred from neighbouring sites
*wR*(*F*^2^) = 0.076	*w* = 1/[σ^2^(*F*_o_^2^) + (0.0436*P*)^2^ + 0.2892*P*] where *P* = (*F*_o_^2^ + 2*F*_c_^2^)/3
*S* = 1.02	(Δ/σ)_max_ = 0.001
2368 reflections	Δρ_max_ = 0.25 e Å^−^^3^
154 parameters	Δρ_min_ = −0.32 e Å^−^^3^
0 restraints	

### Special details

**Table:**

Geometry. All e.s.d.'s (except the e.s.d. in the dihedral angle between two l.s. planes) are estimated using the full covariance matrix. The cell e.s.d.'s are taken into account individually in the estimation of e.s.d.'s in distances, angles and torsion angles; correlations between e.s.d.'s in cell parameters are only used when they are defined by crystal symmetry. An approximate (isotropic) treatment of cell e.s.d.'s is used for estimating e.s.d.'s involving l.s. planes.
Refinement. Refinement of *F*^2^ against ALL reflections. The weighted *R*-factor *wR* and goodness of fit *S* are based on *F*^2^, conventional *R*-factors *R* are based on *F*, with *F* set to zero for negative *F*^2^. The threshold expression of *F*^2^ > σ(*F*^2^) is used only for calculating *R*-factors(gt) *etc*. and is not relevant to the choice of reflections for refinement. *R*-factors based on *F*^2^ are statistically about twice as large as those based on *F*, and *R*- factors based on ALL data will be even larger.

### Fractional atomic coordinates and isotropic or equivalent isotropic displacement parameters (Å^2^)

**Table:**

	*x*	*y*	*z*	*U*_iso_*/*U*_eq_	
Br1	0.70444 (3)	0.11259 (2)	0.71170 (3)	0.06222 (13)	
O1	0.66345 (15)	0.35583 (12)	0.61783 (15)	0.0391 (4)	
O2	0.7122 (2)	0.73040 (14)	0.57865 (18)	0.0640 (5)	
O3	0.84162 (18)	0.72842 (13)	0.82550 (18)	0.0529 (4)	
C1	0.6947 (2)	0.24005 (18)	0.8181 (2)	0.0424 (5)	
H1	0.6541	0.2205	0.8820	0.051*	
C11	0.6034 (2)	0.32101 (18)	0.7079 (2)	0.0404 (5)	
H11	0.5125	0.2915	0.6480	0.048*	
C13	0.7789 (3)	0.8290 (2)	0.7886 (3)	0.0547 (7)	
H13A	0.8441	0.8848	0.8377	0.066*	
H13B	0.7043	0.8344	0.8138	0.066*	
C4	0.7757 (2)	0.42229 (17)	0.7136 (2)	0.0348 (5)	
C2	0.8358 (2)	0.2838 (2)	0.9109 (3)	0.0479 (6)	
H2A	0.8985	0.2259	0.9566	0.057*	
H2B	0.8345	0.3275	0.9878	0.057*	
C7	0.7737 (2)	0.66148 (18)	0.7019 (2)	0.0439 (5)	
C5	0.8222 (3)	0.48727 (18)	0.6187 (3)	0.0482 (6)	
H5A	0.8906	0.4482	0.6038	0.058*	
H5B	0.7453	0.4997	0.5239	0.058*	
C3	0.8863 (2)	0.34948 (19)	0.8204 (3)	0.0439 (5)	
H3A	0.9638	0.3916	0.8854	0.053*	
H3B	0.9165	0.3025	0.7663	0.053*	
C9	0.7113 (2)	0.49168 (18)	0.7904 (2)	0.0371 (5)	
H9	0.7779	0.5045	0.8922	0.044*	
C8	0.6607 (2)	0.59613 (19)	0.7111 (3)	0.0463 (6)	
H8A	0.6205	0.6371	0.7613	0.056*	
H8B	0.5900	0.5822	0.6130	0.056*	
C6	0.8809 (3)	0.59178 (19)	0.6909 (3)	0.0510 (6)	
H6A	0.9192	0.6282	0.6350	0.061*	
H6B	0.9540	0.5790	0.7881	0.061*	
C12	0.7281 (3)	0.8340 (2)	0.6265 (3)	0.0637 (7)	
H12A	0.6419	0.8713	0.5805	0.076*	
H12B	0.7934	0.8703	0.6032	0.076*	
C10	0.5940 (2)	0.4216 (2)	0.7848 (3)	0.0467 (6)	
H10A	0.6061	0.4061	0.8825	0.056*	
H10B	0.5065	0.4560	0.7300	0.056*	

### Atomic displacement parameters (Å^2^)

**Table:**

	*U*^11^	*U*^22^	*U*^33^	*U*^12^	*U*^13^	*U*^23^
Br1	0.0895 (3)	0.04123 (17)	0.0633 (2)	−0.00573 (13)	0.04119 (18)	−0.00022 (12)
O1	0.0431 (9)	0.0423 (8)	0.0294 (8)	−0.0076 (7)	0.0142 (7)	−0.0022 (6)
O2	0.0890 (14)	0.0478 (10)	0.0373 (10)	−0.0019 (10)	0.0129 (10)	0.0008 (8)
O3	0.0545 (10)	0.0443 (9)	0.0425 (9)	0.0021 (8)	0.0068 (8)	−0.0056 (7)
C1	0.0489 (14)	0.0453 (13)	0.0364 (12)	−0.0032 (10)	0.0223 (11)	0.0011 (10)
C11	0.0321 (12)	0.0482 (13)	0.0380 (13)	−0.0066 (10)	0.0134 (10)	0.0023 (10)
C13	0.0602 (17)	0.0420 (14)	0.0590 (17)	−0.0012 (12)	0.0246 (14)	−0.0077 (12)
C4	0.0335 (12)	0.0383 (11)	0.0327 (12)	−0.0032 (9)	0.0153 (10)	−0.0047 (10)
C2	0.0453 (14)	0.0514 (14)	0.0364 (13)	0.0075 (11)	0.0094 (11)	0.0049 (11)
C7	0.0488 (14)	0.0384 (12)	0.0372 (13)	−0.0014 (11)	0.0132 (11)	−0.0032 (10)
C5	0.0584 (16)	0.0430 (13)	0.0569 (15)	−0.0040 (11)	0.0382 (13)	−0.0051 (11)
C3	0.0313 (12)	0.0458 (12)	0.0504 (14)	0.0011 (10)	0.0149 (11)	−0.0047 (11)
C9	0.0325 (12)	0.0443 (12)	0.0337 (12)	0.0026 (9)	0.0144 (10)	−0.0042 (10)
C8	0.0370 (12)	0.0462 (14)	0.0493 (15)	0.0067 (10)	0.0140 (11)	−0.0024 (11)
C6	0.0516 (15)	0.0453 (14)	0.0621 (16)	−0.0098 (11)	0.0311 (13)	−0.0067 (12)
C12	0.075 (2)	0.0490 (16)	0.0596 (17)	0.0066 (14)	0.0237 (15)	0.0063 (13)
C10	0.0399 (13)	0.0549 (14)	0.0508 (14)	0.0033 (11)	0.0255 (12)	0.0035 (12)

### Geometric parameters (Å, º)

**Table:**

Br1—C1	1.979 (2)	C2—H2B	0.9700
O1—C11	1.430 (3)	C7—C6	1.519 (3)
O1—C4	1.449 (3)	C7—C8	1.533 (4)
O2—C12	1.384 (3)	C5—C6	1.511 (3)
O2—C7	1.430 (3)	C5—H5A	0.9700
O3—C13	1.416 (3)	C5—H5B	0.9700
O3—C7	1.424 (3)	C3—H3A	0.9700
C1—C2	1.512 (3)	C3—H3B	0.9700
C1—C11	1.519 (3)	C9—C8	1.520 (3)
C1—H1	0.9800	C9—C10	1.547 (3)
C11—C10	1.528 (3)	C9—H9	0.9800
C11—H11	0.9800	C8—H8A	0.9700
C13—C12	1.498 (4)	C8—H8B	0.9700
C13—H13A	0.9700	C6—H6A	0.9700
C13—H13B	0.9700	C6—H6B	0.9700
C4—C3	1.524 (3)	C12—H12A	0.9700
C4—C5	1.531 (3)	C12—H12B	0.9700
C4—C9	1.552 (3)	C10—H10A	0.9700
C2—C3	1.529 (3)	C10—H10B	0.9700
C2—H2A	0.9700		
			
C11—O1—C4	104.04 (15)	C4—C5—H5A	109.5
C12—O2—C7	109.37 (19)	C6—C5—H5B	109.5
C13—O3—C7	107.58 (18)	C4—C5—H5B	109.5
C2—C1—C11	111.43 (19)	H5A—C5—H5B	108.1
C2—C1—Br1	110.40 (16)	C4—C3—C2	111.99 (18)
C11—C1—Br1	108.80 (15)	C4—C3—H3A	109.2
C2—C1—H1	108.7	C2—C3—H3A	109.2
C11—C1—H1	108.7	C4—C3—H3B	109.2
Br1—C1—H1	108.7	C2—C3—H3B	109.2
O1—C11—C1	110.25 (17)	H3A—C3—H3B	107.9
O1—C11—C10	103.78 (17)	C8—C9—C10	112.51 (19)
C1—C11—C10	110.77 (19)	C8—C9—C4	111.12 (18)
O1—C11—H11	110.6	C10—C9—C4	102.94 (18)
C1—C11—H11	110.6	C8—C9—H9	110.0
C10—C11—H11	110.6	C10—C9—H9	110.0
O3—C13—C12	103.1 (2)	C4—C9—H9	110.0
O3—C13—H13A	111.2	C9—C8—C7	113.1 (2)
C12—C13—H13A	111.2	C9—C8—H8A	108.9
O3—C13—H13B	111.2	C7—C8—H8A	108.9
C12—C13—H13B	111.2	C9—C8—H8B	108.9
H13A—C13—H13B	109.1	C7—C8—H8B	108.9
O1—C4—C3	107.08 (17)	H8A—C8—H8B	107.8
O1—C4—C5	107.98 (17)	C5—C6—C7	111.8 (2)
C3—C4—C5	113.22 (18)	C5—C6—H6A	109.3
O1—C4—C9	103.17 (16)	C7—C6—H6A	109.3
C3—C4—C9	112.15 (18)	C5—C6—H6B	109.3
C5—C4—C9	112.49 (18)	C7—C6—H6B	109.3
C1—C2—C3	111.68 (18)	H6A—C6—H6B	107.9
C1—C2—H2A	109.3	O2—C12—C13	106.1 (2)
C3—C2—H2A	109.3	O2—C12—H12A	110.5
C1—C2—H2B	109.3	C13—C12—H12A	110.5
C3—C2—H2B	109.3	O2—C12—H12B	110.5
H2A—C2—H2B	107.9	C13—C12—H12B	110.5
O3—C7—O2	105.78 (18)	H12A—C12—H12B	108.7
O3—C7—C6	107.5 (2)	C11—C10—C9	104.20 (17)
O2—C7—C6	111.1 (2)	C11—C10—H10A	110.9
O3—C7—C8	112.2 (2)	C9—C10—H10A	110.9
O2—C7—C8	108.2 (2)	C11—C10—H10B	110.9
C6—C7—C8	111.80 (19)	C9—C10—H10B	110.9
C6—C5—C4	110.50 (19)	H10A—C10—H10B	108.9
C6—C5—H5A	109.5		

### Hydrogen-bond geometry (Å, º)

**Table:**

*D*—H···*A*	*D*—H	H···*A*	*D*···*A*	*D*—H···*A*
C11—H11···O2^i^	0.98	2.53	3.445 (3)	156
C1—H1···O1^ii^	0.98	2.57	3.471 (3)	153

Symmetry codes: (i) −*x*+1, −*y*+1, −*z*+1; (ii) *x*, −*y*+1/2, *z*+1/2.
